# Draft Genome Sequence of *Haloferax* sp. Strain ATB1, Isolated from a Semi-Arid Region in the Brazilian Caatinga

**DOI:** 10.1128/genomeA.00812-14

**Published:** 2014-08-14

**Authors:** Wendel de Oliveira Castro, Adriana Maria Torres-Ballesteros, Cristina Rossi Nakayama, Itamar Soares Melo, Vivian Helena Pellizari, Artur Silva, Rommel Thiago Jucá Ramos

**Affiliations:** a Institute of Biological Sciences, Federal University of Pará, Belém, PA, Brazil; b Laboratory of Ecology of Micro-organisms, IO, University of São Paulo, São Paulo, Brazil; c Institute of Environmental, Chemical and Pharmaceutical Sciences, Federal University of São Paulo, Diadema, Brazil; d Laboratory of Environmental Microbiology, EMBRAPA Environment, Jaguariúna, São Paulo, Brazil

## Abstract

Organisms in the *Haloferax* genus are extreme halophiles that grow in environments with pH values between 4 and 12, and temperatures between 0°C and 60°C. In the present study, a draft of the first *Haloferax* sp. strain ATB1 genome isolated from the region of Cariri (in Paraíba State, Brazil) is presented.

## GENOME ANNOUNCEMENT

Organisms in the *Haloferax* genus, which belong to the Archaea domain, characteristically inhabit extreme environments. In particular, these organisms can grow in locations with salinities ranging from 10% to 37%, pH values between 4 and 12, and temperatures between 0°C and 60°C (1). For instance, *H. chudinovii* grows at a pH of 6.8, 42°C, and 16% salinity (2). Halophilic species, which are resistant to dehydration, exhibit low levels of cellular macromolecule denaturation and high thermal stability (3). Moreover, these species are resistant to ultraviolet (UV) light because they contain light-driven proton pumps and produce high levels of carotenoids (4). Thus, halophilic organisms can be used as models to help understand the fundamental limits of life (5) and to identify proteins with biotechnological potential.

The *Haloferax* sp. ATB1 specimen examined in the present study was isolated from the soil of the Cariri complex (in Paraíba, Brazil), a portion of the Caatinga biome that is affected by the desertification processes. The isolate was cultured at a pH of 7.4, temperatures ranging from 37°C to 50°C, and a salinity of approximately 25%. The genome was sequenced with the Ion Torrent PGM platform using a mate-paired library (6). A crude data quality analysis was performed using the FastQC program (http://www.bioinformatics.babraham.ac.uk/projects/fastqc/). The data was submitted to quality filter to remove the reads with quality values less than Phred 20.

This approach resulted in 8,480.874 reads, which corresponded to a genomic coverage of 224× based on the size of the reference genome, *H. volcanni* DS2 (4.1 Mb, including the main chromosome [CP001956] and the plasmids pHV1 [CP001957], pHV2 [CP001954], pHV3 [CP001953], and pHV4 [CP001955]).

A hybrid approach that utilized the four assemblers MIRA (7), CLC Genomics Workbench (http://www.clcbio.com/), SeqMan (http://www.dnastar.com), and SPAdes (8) was adopted for assembly; these assemblers produced 20,586, 14,800, 2,455, and 10,475 contigs, respectively. These results were integrated to generate a single set of sequences. This sequence set was subsequently processed using the CISA program (9) to finalize gaps, producing a total of only 120 contigs with an *N*_50_ of 36,538 that totaled 4.1 Mb.

The contigs were subjected to an automatic annotation process using the RAST software (10). This process identified 4,517 coding sequences (CDSs) and 33 RNAs, which had a G+C content of 61%. The examined *Haloferax* sp. ATB1 genome contains 4,223,705 bp.

### Nucleotide sequence accession number.

The genomic sequence obtained in this study has been deposited in the DDBJ/EMBL/GenBank database under accession number JPES00000000.
